# Beam complexity and monitor unit efficiency comparison in two different volumetric modulated arc therapy delivery systems using automated planning

**DOI:** 10.1186/s12885-021-07991-6

**Published:** 2021-03-10

**Authors:** Chengqiang Li, Cheng Tao, Tong Bai, Zhenjiang Li, Ying Tong, Jian Zhu, Yong Yin, Jie Lu

**Affiliations:** Department of Radiation Oncology Physics, Shandong Cancer Hospital and Institute, Cancer Hospital affiliated to Shandong First Medical University and Shandong Academy of Medical Sciences, Jinan, 250117 China

**Keywords:** Volumetric modulated arc therapy, Beam complexity, Monitor unit efficiency, Auto-planning, Breast cancer, Nasopharyngeal carcinoma

## Abstract

**Background:**

To investigate the beam complexity and monitor unit (MU) efficiency issues for two different volumetric modulated arc therapy (VMAT) delivery technologies for patients with left-sided breast cancer (BC) and nasopharyngeal carcinoma (NPC).

**Methods:**

Twelve left-sided BC and seven NPC cases were enrolled in this study. Each delivered treatment plan was optimized in the Pinnacle^3^ treatment planning system with the Auto-Planning module for the Trilogy and Synergy systems. Similar planning dose objectives and beam configurations were used for each site in the two different delivery systems to produce clinically acceptable plans. The beam complexity was evaluated in terms of the segment area (SA), segment width (SW), leaf sequence variability (LSV), aperture area variability (AAV), and modulation complexity score (MCS) based on the multileaf collimator sequence and MU. Plan delivery and a gamma evaluation were performed using a helical diode array.

**Results:**

With similar plan quality, the average SAs for the Trilogy plans were smaller than those for the Synergy plans: 55.5 ± 21.3 cm^2^ vs. 66.3 ± 17.9 cm^2^ (*p* < 0.05) for the NPC cases and 100.7 ± 49.2 cm^2^ vs. 108.5 ± 42.7 cm^2^ (*p* < 0.05) for the BC cases, respectively. The SW was statistically significant for the two delivery systems (NPC: 6.87 ± 1.95 cm vs. 6.72 ± 2.71 cm, *p* < 0.05; BC: 8.84 ± 2.56 cm vs. 8.09 ± 2.63 cm, *p* < 0.05). The LSV was significantly smaller for Trilogy (NPC: 0.84 ± 0.033 vs. 0.86 ± 0.033, *p* < 0.05; BC: 0.89 ± 0.026 vs. 0.90 ± 0.26, *p* < 0.05). The mean AAV was significantly larger for Trilogy than for Synergy (NPC: 0.18 ± 0.064 vs. 0.14 ± 0.037, *p* < 0.05; BC: 0.46 ± 0.15 vs. 0.33 ± 0.13, *p* < 0.05). The MCS values for Trilogy were higher than those for Synergy: 0.14 ± 0.016 vs. 0.12 ± 0.017 (*p* < 0.05) for the NPC cases and 0.42 ± 0.106 vs. 0.30 ± 0.087 (*p* < 0.05) for the BC cases. Compared with the Synergy plans, the average MUs for the Trilogy plans were larger: 828.6 ± 74.1 MU and 782.9 ± 85.2 MU (*p* > 0.05) for the NPC cases and 444.8 ± 61.3 MU and 393.8 ± 75.3 MU (*p* > 0.05) for the BC cases. The gamma index agreement scores were never below 91% using 3 mm/3% (global) distance to agreement and dose difference criteria and a 10% lower dose exclusion threshold.

**Conclusions:**

The Pinnacle^3^ Auto-Planning system can optimize BC and NPC plans to achieve the same plan quality using both the Trilogy and Synergy systems. We found that these two systems resulted in different SAs, SWs, LSVs, AAVs and MCSs. As a result, we suggested that the beam complexity should be considered in the development of further methodologies while optimizing VMAT autoplanning.

## Background

Currently, volumetric modulated arc therapy (VMAT) is one of the most advanced delivery techniques in radiotherapy. It simultaneously integrates multileaf collimator (MLC) field shape modulation with gantry speed and dose rate variations. With more degrees of freedom during treatment, advanced arc plans provide more flexibility in shaping dose distributions and deliver plans more efficiently than other static beam plan delivery systems [1, 2]. The VMAT technique has become clinically and commercially available via the Trilogy (Varian Medical Systems, Palo Alto, CA, USA) and Synergy (Elekta AB, Stockholm, Sweden) linear accelerators. However, there are many differences between the two machines, such as the method of dose rate control [3], MLC leaf width [4] and jaw tracking technique [5]. Several investigations have been performed to show the dosimetric effects on these system differences.

Studies have suggested that VMAT may be useful at a variety of treatment sites, such as BC [6–8] and NPC [9–12]. As well known, high-quality treatment plans rely on the skills and experience of the dosimetrist, which can vary greatly. Recently, some automatic planning solutions were developed, such as atlas-based planning, ideal dose distribution estimation and template-based optimization [13].

The template-based Auto-Planning module has recently become clinically available in the Pinnacle^3^ radiation therapy treatment planning system (TPS). Several comprehensive studies have been performed to evaluate the benefit of this new autoplanning system [13–16]. The results showed that the Auto-Planning VMAT technique achieves adequate target dose coverage while maintaining low doses to organs-at-risk and therefore reduces the potential for the induction of second malignancy and side effects. Another conclusion from the existing research results is that the Auto-Planning module can generate plans with consistent quality.

Due to the highly choreographed nature of VMAT delivery, many potential sources of error arise, necessitating patient-specific quality assurance (QA) and dosimetric verification techniques [17]. The treatment plan of VMAT requires a compromise between dose conformity (complexity) and deliverability. The creation of a modulation complexity score (MCS) [18] based on plan parameters allows for a quantitative assessment of plan complexity and can provide more information related to dose delivery than simple beam parameters such as monitor units (MUs). Excessive complexity in VMAT plans increases the dose uncertainty, prolongs the treatment time, and increases the susceptibility to changes in patient or target geometry. McNiven et al. [19] performed a study to evaluate the utility of the MCS to evaluate the relationship between the metric and deliverability in IMRT and reported that different clinical treatment sites have an inherent difference in the level of complexity; the average MCS of IMRT plans for the head and neck and for the breast were 0.165 and 0.909, respectively.

However, use of the MCS for the auto-VMAT plan for different treatment sites is still rare. The purpose of this study is to evaluate the beam complexity of two delivery systems optimized by the Auto-Planning module.

## Methods

### Patients

A retrospective analysis was performed on 12 patients with left-sided BC and 7 patients with NPC who were randomly selected from our institution. The prescription dose was 50.0 Gy in 25 fractions for the BC group. There were three different dose levels in patients with NPC, i.e., 54 Gy, 60 Gy, and 66 Gy, for 30 treatment fractions with a simultaneous integrated boost technique.

For BC, the plan objectives were D100% > 100% for the CTV and D100% > 90% for the PTV, with a mean dose for the heart and contralateral breast. V_XGy_ to the ipsilateral lung and body was minimized to keep the dose to the organs-at-risk (OARs) as low as possible by setting higher priority upon avoidance of the contralateral breast, lungs and heart, without compromising the PTV dose coverage, and the maximum hotspots should not exceed 110%.

For NPC, the treatment goals were that 95% of the PTV should receive more than 95% of the prescribed dose and that the maximum dose should be below 110% of the prescription dose. Regarding the OARs, the maximum doses to the brain stem and the spinal cord were set to 54 Gy and 45 Gy, respectively. In addition, at least one side of the parotid glands should receive a mean dose less than 26 Gy, or the volume receiving 30 Gy radiation should be < 50%. The dose to other normal tissues was minimized within a reasonable range without affecting the target coverage.

### Treatment optimization

Treatment planning was performed with the Pinnacle^3^ (V9.10, Philips Radiation Oncology Systems, Fitchburg, WI) treatment planning system. Each of the 19 treatment plans was optimized with a Pinnacle Auto-Planning module for a Varian Trilogy (Varian Medical Systems, Palo Alto, CA) linear accelerator, equipped with a 120 leaf Millennium MLC, and an Elekta Synergy (Elekta Ltd., Crawley, UK) linear accelerator, equipped with a 40 leaf MLCi using 6 MV photons. The Varian Trilogy system can deliver VMAT plans using continuously variable dose rates, while Elekta Synergy can deliver VMAT plans using binned variable dose rates. The Trilogy system was equipped with a Millennium MLC-120, which consisted of two banks of 60 MLC leaves, with the outer 20 and inner 40 on each side having widths of 10 mm and 5 mm, respectively. Overtravel distance of Millennium MLC-120 was 15.0 cm. The Synergy system was equipped with MLCs comprising 40 leaf pairs with a projected leaf width of 10 mm at the isocenter which can overtravel the central axis by 12.5 cm. In addition, Synergy provides a jaw tracking technique, while Trilogy does not have the jaw tracking capability.

The NPC VMAT plans in this study used two full arcs with gantry angles of 181°–179° for a CW rotation and 179°–181° for a CCW rotation, and the collimator angles were set to 15° and 345° to avoid possible overlapping tongue and groove effects. The BC VMAT plans consisted of two short partial arcs. Each arc consisted of a 40° gantry rotation, irradiating between gantry angles 296°–336° and 104°–144° for a CW rotation with collimator angles of 5°–10° to decrease the volume of low dose spread to healthy tissue based on our experience. The dose calculations were performed using Pinnacle’s collapsed cone convolution superposition algorithm, with a gantry spacing resolution of 2° and a dose voxel size of 0.3 × 0.3 × 0.3 cm^3^.

The Auto-Planning module requires the user to define a template with prioritized optimization goals for PTV coverage and dose limits for OARs. These prioritized optimization goals are used by the Auto-Planning engine to formulate optimization objectives. Multiple optimization loops iteratively reformulate and adjust the optimization objectives to meet the criteria defined in the template. During the optimization process, the optimization of the target coverage has higher priority than does dose reduction to the OARs. After the auto-optimization, the treatment plans were fine-tuned by an expert planner to adapt the PTV coverage and OAR protection as much as possible by the two delivery systems.

### Complexity scores

To optimize the arc delivery treatment plans, the treatment planning system uses multiple control points (CPs) to represent the arc plan. A CP refers to the instantaneous configuration at a point in time, and ‘segment’ refers to the duration between CPs, although the two may be used synonymously. Once the treatment plan is completed for a patient, the plans are extracted from the treatment planning system as RTPlan files. RTPlan files contain information on the gantry angle, MLC configuration, jaw position, and MU for each CP. As the main part of our work, the beam complexity scores and dose-volumetric parameters were calculated by an in-house software developed in MATLAB (Version 2010b, MathWorks, Natick, MA).

The complexity scores include the MU, segment width (SW), segment area (SA), leaf sequence variability (LSV), aperture area variability (AAV) and modulation complexity score (MCS). Based on Mcniven et al. [19] and Rajasekaran et al. [20], the beam complexity scores listed above and the dose-volumetric parameters were calculated by an in-house software. The SW and SA for a given segment at a certain gantry angle are as described by Eqs. (1) and (2), respectively, where pos is the coordinate of the leaf position and N is the number of in-field moving leaves inside the jaw position. N is the total number of leaf pairs, and n is the index number of leaf pairs. LeafWidth_n_ is the width of the nth leaf pair. ${\mathrm{SA}}_{{\mathrm{segment}}_{\mathrm{i}}}$ can be roughly understood as the area of a beam segment. The leaves that remained closed during treatment were not considered.

1 $$ {\mathrm{SW}}_{{\mathrm{segment}}_{\mathrm{i}}}=\max \left({{\mathrm{pos}}_{\mathrm{n}\in \mathrm{N}}}_{\mathrm{left}\ \mathrm{bank}}-{{\mathrm{pos}}_{\mathrm{n}\in \mathrm{N}}}_{\mathrm{right}\ \mathrm{bank}}\right), $$

2 $$ {\mathrm{SA}}_{{\mathrm{segment}}_{\mathrm{i}}}={\sum}_{\mathrm{n}=1}^{\mathrm{N}}\left({\left\langle {\mathrm{pos}}_{\mathrm{n}}\right\rangle}_{\mathrm{left}\kern0.17em \mathrm{bank}}\hbox{-} {\left\langle {\mathrm{pos}}_{\mathrm{n}}\right\rangle}_{\mathrm{right}\kern0.17em \mathrm{bank}}\right)\times {\mathrm{LeafWidth}}_{\mathrm{n}}. $$

The MCS and its parameters for each VMAT plan were determined by the following formulae:

3 $$ {\mathrm{pos}}_{\mathrm{max}}\left({\mathrm{segment}}_{\mathrm{i}}\right)={\left\langle \max \left({\mathrm{pos}}_{\mathrm{n}\in \mathrm{N}}\right)-\min \left({\mathrm{pos}}_{\mathrm{n}\in \mathrm{N}}\right)\right\rangle}_{\mathrm{MLC}\ \mathrm{bank}} $$

4 $$ {\mathrm{LSV}}_{{\mathrm{segment}}_{\mathrm{i}}}={\left\langle \frac{\sum_{\mathrm{n}=1}^{\mathrm{N}-1}\left({\mathrm{pos}}_{\mathrm{max}}-\left|\left({\mathrm{pos}}_{\mathrm{n}}-{\mathrm{pos}}_{\mathrm{n}+1}\right)\right|\right)}{\left(\mathrm{N}-1\right)\times {\mathrm{pos}}_{\mathrm{max}}}\right\rangle}_{\mathrm{left}\ \mathrm{bank}}\times {\left\langle \frac{\sum_{\mathrm{n}=1}^{\mathrm{N}-1}\left({\mathrm{pos}}_{\mathrm{max}}-\left|\left({\mathrm{pos}}_{\mathrm{n}}-{\mathrm{pos}}_{\mathrm{n}+1}\right)\right|\right)}{\left(\mathrm{N}-1\right)\times {\mathrm{pos}}_{\mathrm{max}}}\right\rangle}_{\mathrm{right}\ \mathrm{bank}} $$

5 $$ {\mathrm{AAV}}_{{\mathrm{segment}}_{\mathrm{i}}}=\frac{\sum_{\mathrm{n}=1}^{\mathrm{N}}\left({\left\langle {\mathrm{pos}}_{\mathrm{n}}\right\rangle}_{\mathrm{left}\ \mathrm{bank}}-{\left\langle {\mathrm{pos}}_{\mathrm{n}}\right\rangle}_{\mathrm{right}\ \mathrm{bank}}\right)\times {\mathrm{LeafWidth}}_{\mathrm{n}}}{\sum_{\mathrm{n}=1}^{\mathrm{N}}\left({\left\langle {\mathrm{pos}}_{\mathrm{n}}\right\rangle}_{\mathrm{left}\ \mathrm{bank}\in \mathrm{arc}}-{\left\langle {\mathrm{pos}}_{\mathrm{n}}\right\rangle}_{\mathrm{right}\ \mathrm{bank}\in \mathrm{arc}}\right)\times {\mathrm{LeafWidth}}_{\mathrm{n}}}. $$

The parameter LSV was used to characterize the variation in segment shape, and the parameter AAV was used to characterize the variation in SA relative to the maximum aperture defined by all the segments. For a given arc of many small segments that are spatially separated from each other, the values of the LSV and AAV decrease.

To summarize the influence of the LSV, AAV and MU, the MCS for a given arc is calculated based on the scores above, as described in Eq. (6):

6 $$ {\mathrm{MCS}}_{\mathrm{arc}}={\sum}_{\mathrm{i}=1}^{\mathrm{I}\hbox{-} 1}\left[\frac{{\mathrm{LSV}}_{{\mathrm{segment}}_{\mathrm{i}}}+{\mathrm{LSV}}_{{\mathrm{segment}}_{\mathrm{i}+1}}}{2}\times \frac{{\mathrm{AAV}}_{{\mathrm{segment}}_{\mathrm{i}}}+{\mathrm{AAV}}_{{\mathrm{segment}}_{\mathrm{i}+1}}}{2}\times {\mathrm{weight}}_{\mathrm{i}}\right]. $$

The MCS calculation, which is based on three parameters, i.e., the segment shape, area and weight, as in the original definition, has a value range from 0 to 1. weight_i_ represents as a percentage of MU delivered between two successive segments and the arc MU. The MCS = 1 means no modulation, and these interpretations suggest that the average MCS score for a treatment site always decreases with increased inherent complexity.

### Dosimetric QA, gantry speed and dose rate comparisons

For dosimetric QA, the VMAT plans were delivered while irradiating a helical diode array dosimeter (ArcCHECK, Sun Nuclear corporation). The measurements were compared to TPS predictions made using the SNC patent software with the following evaluation criteria: 3 mm distance to agreement and 3% (global) dose difference (3 mm/3%), 2 mm/3% and 2 mm/2% with a lower dose exclusion threshold of 10%. A virtual inclinometer was utilized to record the time when the gantry rotated to the angle specified by each control point and the time between control points. The gantry speed and dose rate were calculated using the RTPlan files, acm files and virtual inclinometer, as reported by Wang et al. [21].

### Evaluation

The collected data, represented as the mean ± standard deviation, were analyzed with the SPSS software (version 13.0, Chicago, USA). The differences in the Trilogy and Synergy plans were evaluated by the two-sided Wilcoxon matched-pair signed-rank test. The threshold for statistical significance was set at *p* < 0.05.

## Results

### PTV coverage and OARs sparing

**Fig. 1:**
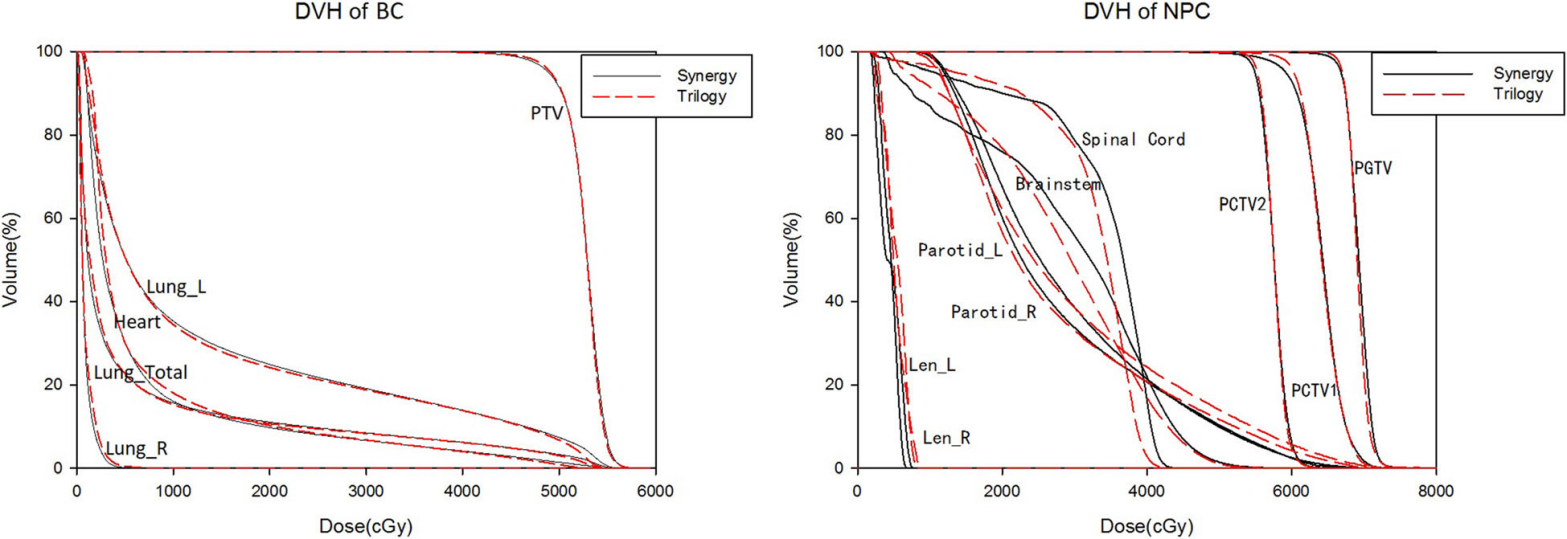
Mean DVH comparison between Trilogy and Synergy plans for BC (left) and NPC (right). Solid line: Synergy; dashed line: Trilogy

The average DVHs for the target volumes and the various OAR comparisons between the Trilogy and Synergy plans for NPC and BC are shown in Fig. 1. All plans sufficiently respected the planning objectives and could be clinically accepted. After post-optimization sequencing by the Auto-Planning module, there was no drastic variation in the dose-volume between the PTVs and OARs.

### MU efficiency

In Table 1, the average PTV and number of MUs are summarized. The average MU among BC patients was 444.8 ± 61.3 for Trilogy and 393.8 ± 75.3 for Synergy. The average MU among NPC patients was 828.6 ± 74.1 for Trilogy and 782.9 ± 85.2 for Synergy.

**Table 1:** Statistical summary of the differences in MU efficiency

Site	Volume	MU		*P* value (Trilogy vs. Synergy)
		Trilogy	Synergy	
NPC	833.4 ± 174.8	828.6 ± 74.1	782.9 ± 85.2	0.128
BC	833.3 ± 198.9	444.8 ± 61.3	393.8 ± 75.3	0.203
*P* value (BC vs. NPC)		0.018	0.017	

### Complexity score comparison

**Fig. 2:**
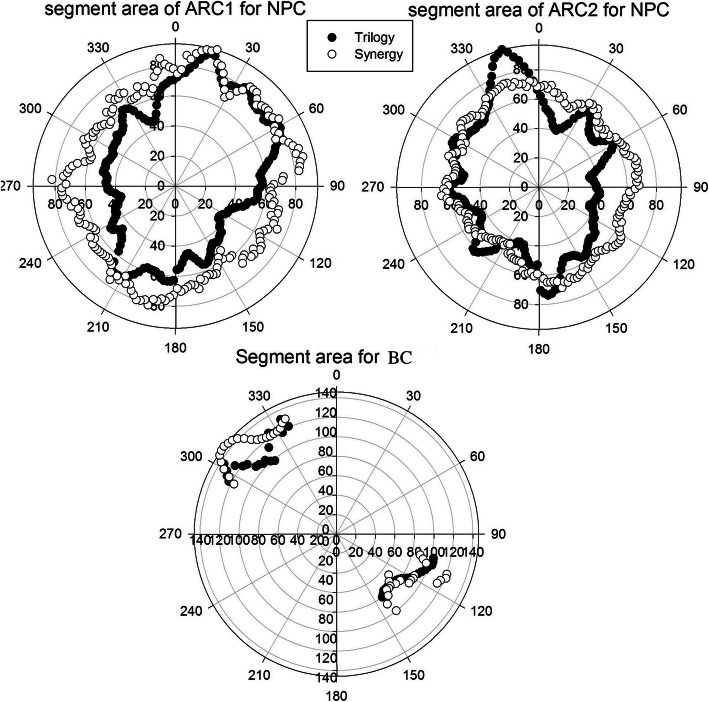
Average SA comparison between Trilogy and Synergy plans for NPC and BC. White circle: Synergy; black circle: Trilogy

**Fig. 3:**
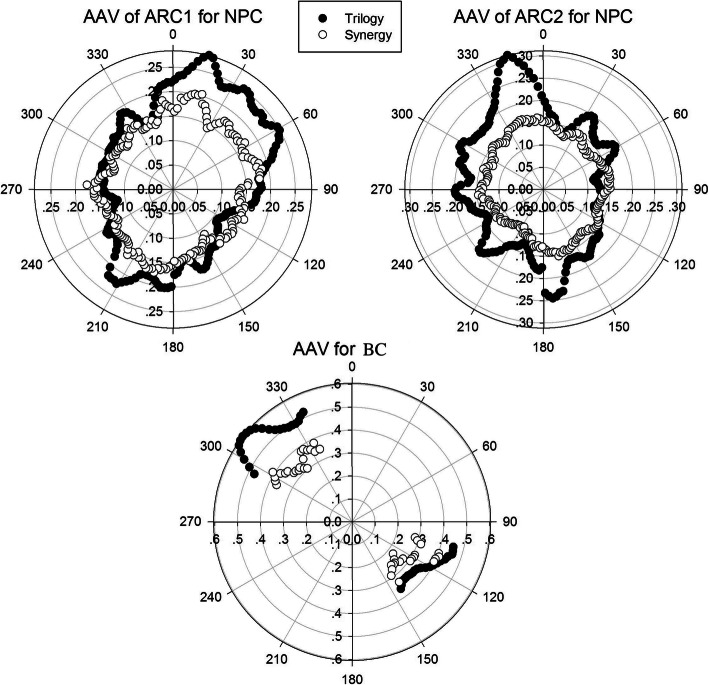
Average AAV comparison between Trilogy and Synergy plans for NPC and BC. White circle: Synergy; black circle: Trilogy

The MCS and its dependent parameters, such as the SA, LSV and AAV, are summarized in Table 2. Significant changes were observed between the Trilogy and Synergy plans in terms of the beam complexity scores for both the NPC and BC plans (*p* < 0.05 for all). Detailed SA and AAV comparisons of the Trilogy and Synergy plans for each control point are given in Figs. 2 and 3.

**Table 2:** Statistical summary of the differences in complexity score

Site	Delivery System	Complexity scores				
		SW (cm)	SA (cm ^2^)	LSV	AAV	MCS
NPC	Trilogy	6.87 ± 1.95	55.5 ± 21.3	0.84 ± 0.033	0.18 ± 0.064	0.14 ± 0.016
	Synergy	6.72 ± 2.71	66.3 ± 17.9	0.85 ± 0.039	0.14 ± 0.037	0.12 ± 0.017
	*P* value	0.000	0.000	0.000	0.000	0.002
BC	Trilogy	8.84 ± 2.56	100.7 ± 49.2	0.89 ± 0.026	0.46 ± 0.15	0.42 ± 0.106
	Synergy	8.09 ± 2.63	108.5 ± 42.7	0.90 ± 0.026	0.33 ± 0.13	0.30 ± 0.087
	*P* value	0.000	0.000	0.031	0.000	0.001

### Dosimetric QA, gantry speed and dose rate comparisons

Table 3 presents a summary of the gantry speed, dose rate and volumetric gamma evaluation (3 mm/3%, 2 mm/3% and 2 mm/2%) results of ArcCHECK. For both Trilogy and Synergy, the gantry speed of the NPC plans was greater than 4°/s. The mean gantry speed of the BC plans was 2.6°/s and 1.1°/s for Trilogy and Synergy, respectively. For the NPC plans, the average passing rates (3 mm/3%) of Trilogy and Synergy were 95.2 and 93.3%, respectively. For the BC plans, the average passing rates (3 mm/3%) of Trilogy and Synergy were 94.5 and 93.7%, respectively.

**Table 3:** Statistical summary of the differences in delivery efficiency and accuracy

Site	Delivery System		Delivery parameters				
		Gantry Speed (deg/s)	Beam-On Time (s)	Dose Rate (MU/min)	Gamma Pass Rate		
					3 mm/3%	2 mm/3%	2 mm/2%
NPC	Trilogy	4.5 ± 0.7	163.2 ± 3.2	242.1 ± 166.7	95.2 ± 1.9	90.3 ± 3.3	83.5 ± 5.6
	Synergy	4.1 ± 0.8	183.2 ± 10.5	244.0 ± 112.5	93.3 ± 0.9	88.2 ± 2.7	77.3 ± 2.2
BC	Trilogy	2.6 ± 1.2	39.3 ± 5.6	531.8 ± 144.4	94.5 ± 2.8	88.5 ± 5.3	81.0 ± 6.4
	Synergy	1.1 ± 0.9	90.4 ± 14.4	258.7 ± 192.9	93.7 ± 2.5	87.9 ± 3.9	81.8 ± 3.9

## Discussion

This work demonstrated that the Pinnacle^3^ Auto-Planning system is able to produce comparable-quality VMAT plans by different delivery systems and is therefore able to successfully handle the geometric and dosimetric variations between NPC and BC sites. Significant differences in the modulation complexity of VMAT plans between Trilogy and Synergy were found.

Dosimetric studies of the autogenerated plans showed excellent target volume coverage and OAR sparing for various target paradigms [13–16]. Hazell et al. [14] compared autogenerated plans with manual head and neck cancer IMRT treatment plans and found that the target coverages in the auto-generated plans were similar to those of the manual plans, though the automatically generated plans had less irradiation of healthy tissue. In contrast to other studies, the primary goal in this study was to generate the “same” plans.

There were several major differences between the Varian Trilogy and Elekta Synergy systems used for delivering VMAT plans. To reduce the variability in the quality of the treatment plans, the dose objectives in the Auto-Planning module can be automatically generated to drive the optimization of a new plan. What is noteworthy is that further user intervention is required to manually set the optimization objectives at the end of the auto-optimization process. Allowing for manual post optimization, the coverage for PTVs and dose constraints for OARs were as similar as possible.

In summary, as shown in Fig. 1, Pinnacle Auto-Planning was able to produce comparable VMAT plans using the Trilogy and Synergy delivery systems for more complex cases (BC and NPC tumor regions). For the NPC plans, differences occurred in the brainstem and spinal cord because the concern was mostly on constraining the maximum dose of to these two organs during optimization. However, the values were still within the tolerance range. In this study, the MLC width and jaw tracking capability were the major parameters influencing the dose distribution. Intuitively, a finer leaf width should result in more conformal target shaping. Lafond et al. [22] showed that beam modulator (4 mm leaf width) and MLCi2 (10 mm leaf width) MLCs from Elekta provided satisfactory dose distributions for head and neck cancer VMAT. OAR sparing was better for the brainstem and spinal cord in the beam modulator. However, the delivery efficiency of VMAT plans was better with MLCi2 in terms of MUs. Our results are in agreement with their study. It is possible that jaw tracking and a larger segment area mainly decrease MLC transmission, which was the likely explanation for the decreased low dose. A reduction in low-dose irradiation of the lungs and lens was found in the Synergy plans in this study. Due to the limited number of cases and the specific site studied, we do not intend the results to be generalizable. A study by Height et al. [23] investigated the effect of different leaf widths from Varian on the treatment of early breast cancer and found no clinically significant differences using 5 mm vs. 10 mm MLC leaf widths. A previous study by French et al. [24] investigated transferring a high-definition MLC (14-32-14 pattern of widths 5-2.5-5 mm) VMAT plan to Millennium MLCs (10-40-10 pattern of widths 10-5-10 mm) and found that a high-definition MLC had a smaller area for a given control point. In addition to differences in leaf numbers and widths, Trilogy and Synergy differ in jaw movement and dose rate bins. Nevertheless, the plan qualities appeared to be equivalent if considered from a clinical perspective for both delivery techniques.

Regarding the treatment MU, the corresponding data listed in Table 1 suggested that although the tumor volumes were similar for NPC and BC, the BC plans had lower MUs than the NPC plans as a result of applying approximately tangential arcs. Synergy required less MUs to treat the same tumor volume, even though there was no drastic variation in the MU value between the two systems for either site. This difference in MU was largely attributed to the fact that large-area segments were more often used in Synergy plans than in Trilogy plans. A similar study in terms of IMRT plans was reported by Qi et al. [25]. As observed from their published study, high MU efficiency was observed in direct aperture optimization (DAO) plans compared with direct machine parameter optimization (DMPO) plans because large-area segments were often used in DAO plans.

Both the Synergy and Trilogy plans had clinically acceptable plan quality, but we observed that the SA was different. The BC plans used a larger SA than that of the NPC plans. Synergy used a larger SA than Trilogy for the same site. The Synergy plans were manifested in the larger segment area and lower MU relative to those of the Trilogy plans. Furthermore, the VMAT auto-generated plans were more sensitive to MLC errors [26].

To assess the beam complexity, as stated in the results, we found significant differences. In this study, the MCSs of the NPC and BC plans were 0.14 and 0.42 for Trilogy and 0.12 and 0.3 for Synergy. Dhanabalan et al. [20] reported that the average MCS for the head and neck VMAT plans was 0.2224. The MCSs for the autogenerated plans were smaller than those for the manual plans, which suggested that the beams were more complex. The auto-generated plans were more modulated, as illustrated by Hansen et al. [13], which might be the reason for the slightly lower pass rate of 97.7% in the ArcCHECK measurements. The question of the relationship between plan complexity and gamma index analysis of delivery accuracy was not included in this study. Regarding gantry rotation speed variations, both Trilogy and Synergy can rotate at high gantry speeds when delivering full-arc NPC plans. However, for the limited angle BC VMAT plans, the gantry speeds of Trilogy and Synergy were different. As shown in Table 3, the Trilogy gantry speed was half that of NPC with a higher dose rate, while Synergy reduced the gantry speed to 1/4 of NPC for BC. Again, it was possible to deliver a VMAT plan more quickly with Trilogy than with Synergy, which was comparable to values reported in other studies with the finding of Osborn et al. [27]. It is noteworthy that the gantry speed and dose rate were calculated from one control point to the next control point. A virtual inclinometer for recording the gantry angle is an effective way to record the time between control points with demonstrated accuracy and high reproducibility [21].

However, much effort has been devoted to analyzing correlations using different patient-specific QA phantoms. Crowe et al. [28] found that the ‘small aperture score’ provided threshold values that successfully distinguished deliverable treatment plans from plans that did not pass QA using a MapCheck2 diode array. Li et al. [29] found that planning parameters such as the average leaf travel and average field indicated a correlation between the plan complexity and the passing rate using an ArcCHECK diode array. Dhanabalan et al. [20] studied the correlation between the MCS and gamma analysis results and indicated that the MCS of a plan has a weak correlation with the planar and volumetric gamma analysis passing rates using the Octavius4D phantom. Knowledge of this relationship will be further accumulated using our MatrixX and ArcCHECK phantoms. This study demonstrates that treatment techniques differ in terms of the treatment MU and MCS. Mcgarry et al. [30] suggested that the MCS was most suitable for inclusion within the cost function to limit complexity in IMRT optimization. VMAT plans, which are less complex, also have higher probabilities of yielding accurate dosimetric results. Further investigations are in progress to confirm the relationship between complexity scores and delivery prediction errors. In general, the current TPSs use optimization algorithms to balance competing dose volume objectives and ignore the complexity. From our viewpoint, when plans are comparable in terms of doses to OARs and PTV coverage, a less complex plan is preferred before it is sent for QA. When 2 plans are comparable in plan quality between two machines, a plan that has a higher prediction gamma rate is preferred. When the plans are complex or have a lower prediction gamma rate, increasing the number of ARCs or CPs can be performed to decrease plan complexity by increasing the number of degrees of freedom. From the planner’s point of view, the plan complex parameters, as a part of the cost function, can be used to guide the plan parameter setting and optimization in a TPS. Finally, the monitoring of complexity allows better consistency in the treatment planning progress.

## Conclusions

In conclusion, all VMAT plans using Trilogy or Synergy implemented in the Pinnacle Auto-Planning module were clinically acceptable and comparable in terms of PTV coverage and OAR sparing for NPC and BC. Differences in the SA, SW, LSV, AAV and MCS, which should be considered in auto-generated plan design, were observed. In practice, this design can be implemented as a function in TPSs in the future.

## Data Availability

All data generated or analyzed during this study are included in this published article.

## Abbreviations

VMAT: Volumetric modulated arc therapy
MU: Monitor unit
BC: Breast cancer
NPC: Nasopharyngeal carcinoma
SA: Segment area
SW: Segment width
LSV: Leaf sequence variability
AAV: Aperture area variability
MCS: Modulation complexity score
MLC: Multi-leaf collimator
TPS: Treatment planning system
QA: Quality assurance
DMPO: Machine parameter optimization
DAO: Direct aperture optimization; OARs: organ at risks
